# Editorial: Cardio-oncology and reverse cardio-oncology: the manifold interconnections between heart failure and cancer

**DOI:** 10.3389/fphys.2023.1205810

**Published:** 2023-05-09

**Authors:** Alessandra Ghigo, Wouter C. Meijers, June-Wha Rhee, Pietro Ameri

**Affiliations:** ^1^ Department of Molecular Biotechnology and Health Sciences, Molecular Biotechnology Center “Guido Tarone”, University of Torino, Torino, Italy; ^2^ Department of Cardiology, Erasmus MC, Rotterdam, Netherlands; ^3^ Department of Medicine, City of Hope National Medical Center, Duarte, CA, United States; ^4^ Department of Internal Medicine, University of Genova, Genova, Italy; ^5^ Cardiovascular Disease Unit, Cardiac, Thoracic and Vascular Department, IRCCS Ospedale Policlinico San Martino—IRCCS Italian Cardiology Network, Genova, Italy

**Keywords:** cardio-oncology, cancer, cardiovascular disease, anticancer therapies, cardiotoxicity

Cardiovascular disease (CVD) and cancer are increasingly recognized as two intimately interconnected conditions, with one predisposing to the other, with a continuous interplay. It is now well established that cancer patients have significantly higher cardiovascular mortality risk than the general population, mostly because of the cardiotoxic side effects of anticancer treatments. In this regard, understanding and tackling cancer therapy-related cardiotoxicity (CTRC) represents the major goal of the branch of cardiology known as cardio-oncology (Lyon et al., 2022).

CTRC remains nowadays a highly relevant Research Topic as treatments with established cardiotoxicity, but also high efficacy, are still widely used, and new anticancer therapies with potential cardiovascular (CV) side effects are continuously introduced in clinical practice. In this Research Topic, the review article by Montisci et al. provided an overview of the CV complications of the most feared oncological treatments, i.e., those requiring admission to the intensive care unit, including but not limited to anthracyclines and anti-HER2 monoclonal antibodies. The review article by Madonna extended this summary by discussing the CV side effects of proteasome inhibitors and tyrosine kinase inhibitors (TKIs), two highly effective treatments for hematological malignancies. Notably, the CV toxicity of these therapies renders these tumor types difficult to treat in the elderly with CV comorbidities, requiring either discontinuation or reduction of drug dosage, with increased likelihood of tumor relapse. The authors then elaborated on the mechanistic basis of the CV side effects of TKIs, suggesting that these drugs could negatively impact the reparative and regenerative properties of the different cell types of the CV system.

If earlier studies proposed a similar mechanism to explain anthracycline cardiotoxicity, the current notion is that the CV side effects of this class of anticancer therapeutics is multi-factorial (Sawicki et al., 2021). In this regard, the original study by Robinson et al. further dissected the molecular basis underlying the late and long-lasting cardiotoxicity that can ensue from a short-termed treatment with anthracyclines. RNA-sequencing of human endomyocardial biopsies, mass spectrometry of genomic DNA and proof-of concept *in vitro* experiments revealed loss of DNA methylation. The authors thus concluded that long-lasting epigenetic modifications resulting from anthracycline treatment are key determinants of late-onset cardiotoxicity. Whether changes in DNA methylation could be associated with a biomarker or used to pinpoint patients at risk for early or late cardiotoxicity remains to be determined by future studies.

Conversely, growing evidence suggests a role of germline genetic variants in mediating the risk of CTRC and the possibility of leveraging these variants to identify patients at risk for CTRC and to guide treatment decisions as well as monitoring and management strategies. In this respect, Yang et al. performed a meta-analysis of 41 studies investigating the relationship between genetic variations and CTRC, with a primary focus on anthracycline-based and HER2-targeted therapies. Out of 14 single nucleotide polymorphisms that were consistently detected in the 41 studies, 6 variants were strongly associated with the increased risk for CTRC. Of note, these variants were anticipated to affect the activity of drug transporters, thus being responsible for drug accumulation and/or excessive reactive oxygen species generation, leading to exaggerated cardiotoxicity. Nevertheless, the pathogenic and mechanistic roles of these variants in mediating cardiotoxicity warrant further investigations.

While acknowledging the importance of CV toxicity of anticancer therapies, contemporary cardio-oncology has widened its focus, since mounting evidence indicates that CVD and cancer are linked to one another at multiple levels (de Boer et al., 2020). The nascent field of reverse cardio-oncology has started to reveal the biological processes shared by cancer and heart failure (HF), such as chronic systemic inflammation and clonal hematopoiesis of indeterminate potential (Ameri et al., 2023). Shared risk factors, such as obesity, also contribute to the interconnection between CVD and HF and represent actionable therapeutic targets. In this Research Topic, Guha et al. highlighted a further layer of complexity in the interplay between risk factors, CVD, and cancer, proposing socio-economic disparity and ancestry as mediators of obesity-driven higher predisposition to CVD and cancer in African Americans (AA) compared to Caucasians. On the one hand, socio-economic disparity hinders access to a healthy lifestyle among AA while, on the other hand, polymorphisms of two genes, APOL1 and ACKR1, were found more frequent in AA than in other ethnicities, suggesting the existence of genetic determinants. Although ACKR1 is a strong attractant of monocytes and could account for enhanced monocyte mobilization and activation in AA, further studies are required to clarify its clinical relevance.

Another cellular process strictly intertwined with inflammation and that could underlie the occurrence of both cancer and CVD is senescence. The review article by Banerjee et al. discussed how the conventional concept of senescence as the mediator of cell cycle arrest has been progressively replaced by the view according to which senescent cells can remain metabolically active and secrete pro-inflammatory mediators known as senescence-associated secretory phenotype (SASP). The article provided an overview of recent evidence suggesting that SASP can be at the intersection of CVD and cancer, suggesting the use of senolytics as an approach for the treatment of both diseases.

Irrespective of common underlying pathways, the interrelation between CVD and cancer is confirmed by the prognostic value that biomarkers classically associated with one condition also play in the other one. While it was previously shown that increased concentrations of established markers of CVD portend a higher risk of cancer incidence and mortality (Jovani et al., 2022), Bracun et al. now reported that 6 tumor biomarkers also predicted CVD and CV mortality in more than 8,000 subjects enrolled in the Prevention of Renal and Vascular End-stage Disease (PREVEND) study, which was originally conducted to assess renal disease and CVD in a large cohort sample in Netherlands. The strongest associations were for CEA and CYFRA 21-1 and, interestingly, differences were observed between males and females, which are worth of additional investigation.

In conclusion, this Research Topic highlights that CVD and cancer are interlinked by shared genetic and non-genetic risk factors, and common mechanistic basis, like inflammation and senescence. Interestingly, anticancer therapies may exacerbate CVD in cancer patients by impinging on the same molecular processes, which could thus represent actionable targets to prevent CTRC.
